# Development of myocarditis and pericarditis after COVID-19 vaccination in adult population: A systematic review

**DOI:** 10.1016/j.amsu.2022.103486

**Published:** 2022-03-11

**Authors:** Maurish Fatima, Huzaifa Ahmad Cheema, Muhammad Huzaifa Ahmed Khan, Hafsa Shahid, Muhammad Saad Ali, Umer Hassan, Muhammad Wahaj Murad, Muhammad Aemaz Ur Rehman, Hareem Farooq

**Affiliations:** a Department of Medicine, King Edward Medical University, Lahore, Punjab, Pakistan; b Shanxi Medical University Yuci District, Jin Zhong City, Shanxi province, China

**Keywords:** COVID-19, Myocarditis, Pericarditis, Vaccine, COVID-19, coronavirus disease 19

## Abstract

**Objectives:**

A clear temporal relationship between myocarditis and pericarditis after COVID-19 vaccination has led to the belief that the vaccine may act as a trigger for these cardiologic complications. The aim of this systematic review is to explore the incidence, clinical presentation, management, and association between them.

**Methods:**

We conducted a systematic literature search on Cochrane, MEDLINE, and EMBASE as per guidelines of PRISMA (Preferred Reporting Items for Systematic Reviews). A total of 41 case reports and case series describing 97 patients, and 5 original articles describing 15,585,309 participants were selected as part of this review.

**Results:**

Of the 97 reported cases describing vaccine-associated myocarditis/pericarditis, 67 (69%) patients received Pfizer-BioNTech and 25 (25.7%) received Moderna. The mean onset of symptoms after vaccine administration was 3.8 ± 4.5 days with three-quarters developing symptoms after the second dose. Chest pain (n = 88, 90%) and fever (n = 33, 34%) were the most common presenting complaints. Out of 97, 80 (82.5%) patients recovered while 4 (4.1%) patients expired. The pooled incidence of myocarditis and pericarditis extrapolated from original studies is 0.001% and 0.0004%, respectively. In the original studies, nearly all the cases of myocarditis and pericarditis were mild. Chest pain and fever were the most common presenting symptoms.

**Conclusion:**

Myocarditis and pericarditis after the COVID-19 vaccine have been reported more in young adult males and are most likely to occur after the second dose of mRNA vaccines. The presentation is mild and the majority of the patients recover either completely or partially.

## Highlights

• Myocarditis and pericarditis have been reported after the COVID-19 vaccine.
• It is occurs more commonly in males as compared to females.
• Myocarditis and Pericarditis is more likely to occur after second dose of mRNA vaccine.
• The presentation is mild, and most of the patients recover either completely or partially.
• Autoimmunity (molecular mimicry) is thought to be the major pathological mediator.

## 1 Introduction

Myocarditis is the inflammation of the myocardium that occurs most commonly due to viral illnesses although non-infectious etiologies have also been reported. It is believed that myocarditis and its complications are largely immune-mediated [1]. Myocarditis usually presents with chest pain, which can result from associated pericarditis, or occasionally, from coronary artery spasm. Acute myocarditis is frequently first diagnosed as nonischemic dilated cardiomyopathy in a symptomatic patient [2]. Pericarditis (inflammation of the pericardium) commonly presents with sharp, retrosternal chest pain that is relieved by sitting or leaning forward but gets exacerbated in the supine position, by coughing, and with inspiration [3].

COVID-19, caused by the novel coronavirus SARS-CoV-2, became a public health emergency of international concern (PHEIC) in January 2020 [4]. According to the latest statistics, over 317 million global cases of SARS-CoV-2 have been reported so far. Mass immunization campaigns have been initiated throughout the world as per the World Health Organization (WHO) recommendations. Multiple coronavirus vaccines are currently being administered throughout the world which includes mRNA based vaccines, (i.e. Pfizer-BioNTech, Moderna), recombinant adenoviral vector vaccines (i.e. Johnson & Johnson/Janssen, Oxford-AstraZeneca and Sputnik V), and the inactivated whole viral vaccines (i.e. Sinovac Biotech and Sinopharm) [5]. Given the rapid global spread and increased associated mortality, the emergency use approval was granted to COVID-19 vaccines before the completion of conventional and robust phases of clinical trials [6]. Therefore, some concerns have been raised regarding the safety as well as the efficacy of these vaccines.

Numerous case reports, case series, and retrospective studies have now suggested a possible link between myocarditis and Covid-19 mRNA vaccination. To explore this phenomenon, we planned to conduct a systematic review in which databases would be thoroughly searched to find out all literature available on post-vaccination myocarditis and pericarditis in adults. A compilation of all such cases will alert the physicians about rare but detrimental side-effects of vaccination and enhance their knowledge regarding the likely clinical presentation, prognosis, and management. The timely diagnosis followed by prompt treatment will ultimately lead to improved patient care.

Several other reviews have reported adverse events after COVID-19 vaccination [7]. To date, only one systematic review and meta-analysis evaluating myocarditis following COVID-19 vaccination has been published in the literature [8]. However, the review included a limited number of cases, focused only on mRNA vaccines, and lacked sufficient discussion on underlying pathogenic mechanisms. This indicates the need for a more comprehensive evidence synthesis that includes original articles and updated evidence. This systematic review aims to provide a detailed account of the development of myocarditis and pericarditis following the COVID-19 vaccination, and serves as a guide for researchers for re-evaluation, who may need to take into consideration this side-effect while developing new vaccines.

## 2 Methods

This systematic review is compliant with the Preferred Reporting Items for Systematic review and Meta-Analyses (PRISMA) guidelines and has been registered with The International Prospective Register of Systematic Reviews (PROSPERO: CRD42021276596) [9] (Supplementary file_3).

### 2.1 Search strategy

The systematic literature search was conducted on the following three databases:

MEDLINE (via PubMed), Cochrane, and Embase without any restriction of language, study design, country, and year of publication. The complete search string for PubMed is given in Table 1.

**Table 1:** Search strategy for MEDLINE (PubMed format).

Number	Search terms
#1	sars-cov-2 [All Fields]
#2	“sars-cov-2” [mh]
#3	covid [All Fields]
#4	covid-19 [All Fields]
#5	“covid-19” [mh]
#6	coronavirus [All Fields]
#7	“coronavirus” [mh]
#8	#1 OR #2 OR #3 OR #4 OR #5 OR #6 OR #7
#9	vaccine [All Fields]
#10	“vaccines” [mh]
#11	“vaccination” [mh]
#12	#9 OR #10 OR #11
#13	#8 AND #12
#14	“COVID-19 Vaccines/adverse effects” [mh]
#15	#13 OR #14
#16	myocarditis [All Fields]
#17	“myocarditis” [mh]
#18	pericarditis [All Fields]
#19	“pericarditis” [mh]
#20	#16 OR #17 OR #18 OR #19
#21	#15 AND #20

### 2.2 Study selection and data extraction

We considered all the peer-reviewed published studies that included the adult population (>19 years) who developed myocarditis and pericarditis following any type (mRNA, viral vector, and protein subunit) of COVID-19 vaccine. Review articles, editorials, preprints and those original articles that reported other side effects of vaccination but did not discuss myocarditis and pericarditis specifically were excluded. This review only included articles written in English language.

Articles were searched and extracted by two reviewers (M.F and H.A.C), and a third investigator (M.H.A.K) was there to resolve any discrepancies. Identified studies were uploaded to Mendeley and duplicates were removed. Initially, the articles were screened based on title and abstract, after which the full articles were reviewed. The retrieved results are summarized in the form of two tables. One table focuses on the demographics, medical history, and outcomes, whereas the second is based on relevant medical investigations and diagnostic findings. Continuous variables are presented as means ± standard deviations, and categorical variables are presented as absolute values and percentages. Microsoft Excel was used for data extraction and calculations carried out in this study. The references were added through Mendeley.

### 2.3 Quality appraisal

The quality of the included articles was assessed by the Joanna Briggs Institute Critical Appraisal Tool for case reports and case series and the Newcastle-Ottawa Scale quality assessment scale for cohorts (available in Supplementary file_1) [10,11]. Three reviewers (M.F, U.H, M.H.A.K) first independently scored each article and then awarded a consensus score to each. The score report is provided in the supplementary files. Due to large heterogeneity between study designs, study populations, outcomes, and outcome measures, a meta-analysis could not be performed. The systematic review has been self-evaluated through the AMSTAR 2 checklist (available in Supplementary file_2) [12]. As no Randomized Controlled trial was included in the review, the level of compliance with AMSTAR 2 came out to be “moderate”.

## 3 Results

**Fig. 1:**
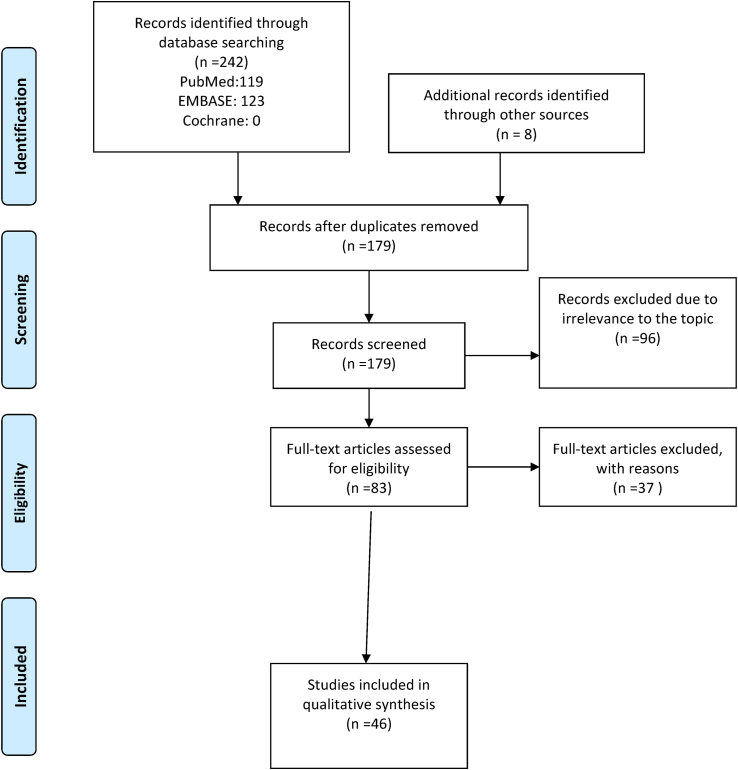
PRISMA flowchart.

The search of three databases identified 250 articles. Seventy-one articles were removed due to duplication and 96 articles were excluded due to irrelevance to the topic. After rigorous screening, 46 articles comprising case series, case reports [2,[13], [14], [15], [16], [17], [18], [19], [20], [21], [22], [23], [24], [25], [26], [27], [28], [29], [30], [31], [32], [33], [34], [35], [36], [37], [38], [39], [40], [41], [42], [43], [44], [45], [46], [47], [48], [49], [50], [51], [52]] and original articles [[53], [54], [55], [56], [57]] were included in our review (Fig. 1).

### 3.1 Case series and case reports

A total of 97 patients were described in 41 case series and case reports. The demographic characteristics, clinical presentation, lab investigations, radiological findings, and treatment of the 97 patients have been elaborated in the form of two tables (Table 2, Table 3).

**Table 2:** Demographics of patients with myocarditis and pericarditis after COVID-19 vaccine.

Sr No	Domain	Author, Year	Country reported	Number of patients		Age(years) Gender M/F	Medical History	Type of Vaccine administered	Myocarditis/Pericarditis	Time between vaccine administration and development of myocarditis/pericarditis
1	Case report	Cimaglia et al. (2021) [29]	Portugal	1		24, Male	E-cigarette smoking	Pfizer-BioNTech	Myocarditis	60 h after second dose
2	Case report	Nguyen et al.(2021) [48]	England	1		20, Male	Not significant	Moderna	Myocarditis	12 h after first dose
3	Case report	Watkins et al. (2021) [14]	USA	1		20, Male	COVID+, Tobacco+	Pfizer-BioNTech	Myocarditis	48 h after second dose
4	Case series	Vidula et al. (2021) [44]	USA	5	Patient-No-1	19, Male,	Not significant	Pfizer-BioNTech	Myocarditis	4 days after second dose
					Patient-No-2	18, Male	Not significant	Moderna	Myocarditis	24 h after second dose
					Patient-No-3	60, Female	Stress cardiopathy	Pfizer-BioNTech	Stress Cardiomyopathy	4 days after second dose
					Patient-No-4	21, Female	Not significant	Pfizer-BioNTech	Pericarditis	3 weeks after first dose
					Patient-No-5	61, female	HTN+	Pfizer-BioNTech	Pericarditis	4 weeks after second dose
5	Case report	Albert et al. (2021) [43]	USA	1		24, Male	Not significant	Moderna	Myocarditis	4 days after second dose
6	Case series	Shaw et al. (2021) [32]	USA	4	Patient,No,1	24, Male	Not significant	Pfizer-BioNTech	Myocarditis	4 days after second dose
					Patient-No-2	31, Female	A history of confirmed COVID+ 7 months ago	Moderna	Myocarditis	25 days after first dose
					Patient-No-3	16, Male	COVID+	Pfizer-BioNTech	Myocarditis	4 days after first dose
					Patient-No-4	17, Female	Not Significant	Pfizer-BioNTech	Myocarditis	2 days after second dose
7	Case report	Habib et al. (2021) [28]	Qatar	1		37, Male	Ex-smoker, alcoholic, HTN + ve	Pfizer-BioNTech	Myocarditis	3 days after second dose
8	Case series	Abbate et al. (2021) [34]	USA	2	Patient-No- 1	27, Male	Downs syndrome + ve,	Pfizer-BioNTech	Fulminant pericarditis	2 days after second dose
					Patient-No-2	34, Female	Not significant	Pfizer-BioNTech	Fulminant myocarditis	9 days after first dose
9	Case series	Mouch et al. (2021) [19]	Israel	6	Patient 1	24, Male	Not significant	Pfizer-BioNTech	Myocarditis	72 h after second dose
					Patient-No-2	20, Male	Not significant	Pfizer-BioNTech	Myocarditis	24 h after second dose
					Patient-No-3	29, Male	Not significant	Pfizer-BioNTech	Myocarditis	48 h after second dose
					Patient-No-4	45, Male	Not significant	Pfizer-BioNTech	Myocarditis	16 days after first dose
					Patient-No-5	16, Male	Not significant	Pfizer-BioNTech	Myocarditis	24 h after second dose
					Patient-No-6	17, Male	Not significant	Pfizer-BioNTech	Myo-pericarditis	72 h after second dose
10	Case report	Ammirati et al.(2021) [45]	Italy	1		56, Male	COVID + ve	Pfizer-BioNTech	Myocarditis	3 days after second dose
11	Case report	Cereda et al. (2021) [51]	Italy	1		21, Male	Not significant	Pfizer-BioNTech	Myocarditis	30 h after second dose
12	Case series	Chamling et al. (2021) [21]	Germany	3	Patient-No-1	68, Female	Tobacco+, CVD+	*AstraZeneca*	Myocarditis	24 h after first dose
					Patient-No-2	25, Male	Smoker + ve,	Pfizer-BioNTech	Myocarditis	10 days after first dose
					Patient-No-3	20, Male	Not significant	Pfizer-BioNTech	Myocarditis	3 days after second dose
13	Case report	D'Angelo et al.(2021) [52]	Italy	1		30, Male	Not significant	Pfizer-BioNTech	Myocarditis	72 h after second dose
14	Case report	Deb et al. (2021) [18]	USA	1		67, Male	HTN+, T2DM, Hyperlipidemia, CAD with CABG, CHD, COPD, GERD	Moderna	Myocarditis	6 h after second dose
15	Case series	Dickey et al. (2021) [30]	USA	6	Patient 1	Male (35–40 year)	Not significant	Pfizer-BioNTech	Myocarditis	4 days after second dose
					Patient 2	Male (16–20 year)	Not significant	Pfizer-BioNTech	Myocarditis	3 days after second dose
					Patient 3	Male (20–25 year)	Not significant	Moderna	Myocarditis	4 days after second dose
					Patient 4	Male (20–25 year)	Not significant	Pfizer-BioNTech	Myocarditis	2 days after second dose
					Patient 5	Male (16–20) year	Not significant	Pfizer-BioNTech	Myocarditis	4 days after second dose
					Patient 6	Male (16–20) year	Not significant	Pfizer-BioNTech	Myocarditis	3 days after second dose
16	Case report	Ehrlich et al. (2021) [36]	Germany	1		40, Male	Not significant	Pfizer-BioNTech	Myocarditis	2 day after first dose
17	Case report	Hasnie et al. (2021) [39]	USA	1		22, Male	COVID + ve	Moderna	Perimyocarditis	3 days after first dose
18	Case series	Hudson et al. (2021) [33]	USA	2	Patient 1	24, Male	Not significant	Pfizer-BioNTech	Myopericarditis	3 days after second dose
					Patient 2	22, Male	Not significant	Pfizer-BioNTech	Myopericarditis	12 h after second dose
19	Case series	Larson et al. (2021) [17]	Italy	8	Patient no 1	22, Male	Not significant	Moderna	Myocarditis	3 days after second dose
					Patient no 2	31, Male	Not significant	Moderna	Myocarditis	3 days after second dose
					Patient no 3	40, Male	COVID + ve,	Pfizer-BioNTech	Myocarditis	2 days after first dose
					Patient no 4	56, Male	Not significant	Pfizer-BioNTech	Myocarditis	3 days after second dose
					Patient 5	26, Male	COVID + ve	Pfizer-BioNTech	Myocarditis	3 days after second dose
					Patient 6	35, Male	Not significant	Pfizer-BioNTech	Myocarditis	2 days after second dose
					Patient 7	21, Male	Not significant	Pfizer-BioNTech	Myocarditis	4 days after second dose
					Patient 8	22, Male	Not significant	Pfizer-BioNTech	Myocarditis	2 days after second dose
20	Case Report	Khogali et al. . 2021 [46]	Qatar	1		29, female	CKD since birth and a background of food allergy	Moderna	Perimyocarditis	10 days after second dose
21	Case Report	Kim et al. . 2021 [16]	Korea	1		24, male	Not significant	Pfizer-BioNTech	Myocarditis	1 day after second dose
22	Case Series	King et al. . 2021 [23]	USA	4	Patient No 1	23, Female	Not significant	Moderna	Myocarditis	5 days after second dose
					Patient No 2	20, Male	Not significant	Moderna	Myocarditis	2 days after second dose
					Patient No 3	29, Male	Not significant	Moderna	Myocarditis	4 days after second dose
					Patient No 4	30, Male	Not significant	Pfizer-BioNTech	Myocarditis	4 days after second dose
23	Case series	Koizumi et al. . 2021 [35]	Japan	2		22, Male	Not significant	Moderna	Myocarditis	2 days after second dose
						27, Male	Not significant	Moderna	Myocarditis	3 days after second dose
24	Case series	Mansour et al. . 2021 [15]	USA	2	Patient No 1	25, Male	Not significant	Moderna	Myocarditis	1 day after second dose
					Patient No 2	21, Female	CVDz + ve(long QT syndrome in siblings)	Moderna	Myocarditis	2 days after second dose
25	Case Report	Matta et al. . 2021 [42]	USA	1		27, Male	Not significant	Pfizer-BioNTech	Myocarditis	3 days after second dose
26	Case Report	Muthukumar et al. . 2021 [27]	USA	1		52, Male	CVD + ve	Moderna	Myocarditis	3 days after second dose
27	Case Report	Nassar et al. . 2021 [26]	USA	1		70, Female	history of multiple sclerosis	Janssen COVID-19 vaccine	Myocarditis	after two days
28	Case Series	Nevet et al. . 2021 [38]	Israel	3		20, 29, and 24 years old men	Not significant	Pfizer-BioNTech	Myocarditis	2 days after second dose
29	Case series	Patel et al., 2021 [31]	USA	Five (5)	Patient no.1	22, Male	History of ADHD+,	Pfizer-BioNTech	Acute myopericarditis	2 days after second dose
					Patient no2	19, Male	History of asthma+	Pfizer-BioNTech	Myopericarditis	1 day after second dose
					Patient no.3	25, Male	Not significant	Moderna	Acute myopericarditis	3 days after second dose
					Patient no.4	37, Male	Not significant	Pfizer-BioNTech	Acute myocarditis	2 days after second dose
					Patient no.5	20, Male	Not significant	Pfizer-BioNTech	Acute myocarditis	3 days after second dose
30	Case series	Rosner et al., 2021 [2]	USA	Seven (7)	Patient no.1	28, Male	Not signficant	Janssen (Ad.26.COV2.S)	Acute myocarditis	5 days after administration of dose
					Patient no.2	39, Male	Not signficant	Pfizer-BioNTech	Acute myocarditis	3 days after second dose
					Patient no.3	39, Male	Not signficant	Moderna	Acute myocarditis	4 days after first dose
					Patient no.4	24, Male	Not signficant	Pfizer-BioNTech	Acute myocarditis	7 days after second dose
					Patient no.5	19, Male	Not signficant	Pfizer-BioNTech	Acute myocarditis	2 days after second dose
					Patient no.6	20, Male,	COVID + history	Pfizer-BioNTech	Acute myocarditis	3 days after second dose
					Patient no. 7	23, Male	COVID + history	Pfizer-BioNTech	Acute myocarditis	3 days after second dose
31	Case report	Singh et al., 2021 [40]	USA	One (1)		24, Male	Ocassional alcoholic	Pfizer-BioNTech	Acute myocarditis	3 days after second dose
32	Case report	Sokolska et al., 2021 [20]	Poland	One (1)		21, Male	Asthma in childhood, history of appendectomy, pollen and pet allergy	mRNA COVID-19 vaccination (Comirnaty, Pfizer)	Acute myocarditis	3 days after first dose
33	Case series	Starekova et al., 2021 [25]	USA	Five (5)	Patient no.1	21, Male	Not signficant	Pfizer-BioNTech	Acute myocarditis	2 days after second dose
					Patient no2	32, Female	Not signficant	Pfizer-BioNTech	Acute myocarditis	3 days after second dose
					Patient no.3	17, Male	Not significant	Pfizer-BioNTech	Acute myocarditis	2 days after second dose
					Patient no.4	18, Male	Not significant	Moderna	Acute myocarditis	3 days after second dose
					Patient no.5	38, Male	Not significant	Moderna	Acute myocarditis	3 days after second dose
34	Case report	Tailor et al., 2021 [47]	USA	One (1)		44, Male	Former smoker, Drug history: Albuterol, Salmetrol-fluticasone	Moderna	Acute myocarditis	4 days after second dose
35	Case report	Ujueta et al., 2021 [37]	USA	One (1)		62, Female	Medical history significant for melanoma status post-surgical resection and treatment with Pembrolizumab over one year prior as well as essential thrombocytosis currently receiving treatment with Anagrelide	Janssen Johnson & John-son (Ad.26.COV2.S)	Lypmhohistiocytic myocarditis	4 days after vaccine
36	Case series	Verma et al., 2021 [41]	USA	Two (2)	Patient no.1	45, Female	Not significant	Pfizer-BioNTech	Fulminant myocarditis	10 days after first dose
					Patient no2	42, Male	Not signficant	Moderna	Fulminant myocarditis	14 days after second dose
37	Case report	*Williams* et al., 2021 [50]	USA	One (1)		34, Male	Not signficant	Moderna	Perimyocarditis	1 day after second dose
38	Case series	Levin et al.(2021) [24]	Israel	7	Patient 1	20, Male	ADHD	Pfizer-BioNTech	Myocarditis	1 day after second dose
					Patient no 2	19, Male	Celiac disease	Pfizer-BioNTech	Myocarditis	1 day after second dose
					Patient No-3	19, male	Allergic asthma	Pfizer-BioNTech	Myocarditis	1 day after second dose
					Patient No-4	22, Male	Not significant	Pfizer-BioNTech	Myocarditis	5 day after second dose
					Patient No-5	24, Male	Not significant	Pfizer-BioNTech	Myocarditis	2 days after second dose
					Patient-No-6	21, Male	Myocarditis 5 years ago	Pfizer-BioNTech	Myocarditis	5 days after second dose
					Patient-No-7	18, Male	Not significant	Pfizer-BioNTech	Myocarditis	2 days after second dose
39	Case report	Patrignani et al. (2021) [22]	Italy	1		56, Male	COVID+ 5 months ago	Pfizer-BioNTech	Myocarditis	4 days after first dose
40	Case report	Sulemankhil et al.(2021) [49]	USA	1		33, Male	History of asthma and sleep apnea	Janssen Johnson & John-son (Ad.26.COV2.S)	Myocarditis	24 h after vaccination
41	Case report	Garcia et al. (2021) [13]	Spain	1		39, Male	History of asthma, autoimmune hypothyroidism, chronic atrophic gastritis, an isolated episode of atrial fibrillation, and recurrent spontaneous pneumothorax with left apical lobectomy	Pfizer-BioNTech	Pericarditis	6 h after second dose

COPD: Chronic Obstructive Pulmonary Disease, CKD: Chronic Kidney Disease, CVD: Cardiovascular Disease, CAD: Coronary Artery Disease, ADHD: Attention Deficit Hyperactivity Disorder, GERD: Gastroesophageal Reflux Disease, COVID: Coronavirus Disease, CHD: Coronary Heart Disease, CABG: Coronary Artery Bypass Grafting, HTN: Hypertension, T2DM: type 2 Diabetes Mellitus.

**Table 3:** Clinical Presentation, Lab investigations and Diagnostic findings in patients with myocarditis and pericarditis after COVID-19 vaccine.

Sr No	Domain	Author, Year	Clinical features	ECG Findings	Echocardiogram findings	Lab Investigations	Treatment	Diagnostic Criteria (CMR imaging findings)	Additional Comments
1	Case report	Cimaglia et al. (2021) [29]	Chest pain exacerbated by deep breathing and supine position	ST elevation in I, II and aVF, mild ST depression in V1 to V3	LVEF 45%	Cardiac troponin T 1204 ng/L, C-reactive protein (1.9 mg/dL)	Anti-inflammatory therapy	Mildly dilated LV with normal EF and no regional kinesis abnormality	Recovered and discharged
2	Case report	Nguyen et al.(2021) [48]	Fever, myalgia, fatigue, and growing mid-sternal burning chest pain without radiation 12 h after vaccine administration	Normal ECG	LVEF = 53–56%	Cardiac troponin T (333 pg/mL). C-reactive protein (19.6 mg/L)	Not mentioned	Subepicardial and intramural LGE in mid and basal inferolateral segment indicating myocardial edema	Recovered and Discharged
3	Case report	Watkins et al. (2021) [14]	Midsternal chest pain radiating to the left side	ST-segment elevations with PR depressions (V5–V6, II, aVF).	LVEF 59%	Troponin increased to a maximum of 108 ng/L.	Colchicine, metoprolol, and ibuprofen.	CMR positive for myocarditis	Recovered and Discharged
4	Case series	Vidula et al. (2021) [44]	Acute substernal chest pressure, dyspnea	Diffuse ST elevations	LVEF: 47%;	NOT significant	Lisinopril and metoprolol succinate	CMR revealed mild hypokinesis of the basal to mid-lateral wall with elevated corresponding T1 value, elevated T2 value and sub-epicardial delayed enhancement in the lateral wall.	Recovered and Discharged
			fever, myalgia, acute substernal chest pain	Diffuse ST elevations	LVEF: 59%,	High-sensitive troponin: 7206 ng/L, CRP: 74.2 mg/L	Discharged on metoprolol succinate and a course of colchicine and ibuprofen.	Subepicardial LGE involving the mid-lateral wall, with corresponding elevated native T1 and T2 values	Recovered and Discharged
			History of the stent in LAD(left anterior descending artery)	Diffuse ST-elevation	LVEF = 44%	Troponin T: 0.129 ng/mL	Metoprolol succinate and lisinopril	Not performed	Recovered and Discharged
			Chest pain that worsened during inspiration and while supine	Not mentioned	LVEF: 60%; pericardial effusion	Troponin T: undetectable.CRP: 72.6 mg/L	Colchicine	Cardiac MRI not performed	Recovered and discharged
			Fever, night sweats, chest discomfort, palpitations	Not mentioned	LVEF: 65%; pericardial effusion	Troponin T: undetectable.CRP: 23.1 mg/dL	Colchicine	Cardiac MRI not performed	Recovered and Discharged
5	Case report	Albert et al. (2021) [43]	Substernal chest pain exacerbated with deep inspiration and supine position	Normal ECG	within normal range, with LVEF within 65%	Elevated troponin I (18.94 ng/mL), elevated C Reactive Protein (26.4 mg/L)	Discharged on Beta blocker	Normal LV size and EF (58%), mid-myocardial and epicardial edema.	Recovered and Discharged
6	Case series	Shaw et al. (2021) [32]	Chest pain	Not mentioned	Not mentioned	Troponin I elevated to 4.963 ng/mL	Not mentioned	CMR demonstrated LVEF = 56%. epicardial edema	No follow-up mentioned
			Chest pain	Not mentioned	Not mentioned	Troponin I elevated to 7.961 ng/mL (normal <0.034 ng/mL)	Not mentioned	CMR demonstrated LVEF = 57%, On T2 mapping, there were skip areas of epicardial edema involving the basal inferior, basal, mid, and apical lateral segments (59 ms–66 ms, normal <55 ms) and nonischemic myocardial injury on native T1 mapping (1117 ms-1137 ms, normal 950 ms-1050 ms). Epicardial fibrosis was observed on LGE imaging and interstitial expansion by extracellular volume fraction mapping (40%–44%, normal <28%).	No follow-up mentioned
			Chest pain	Not mentioned	Not mentioned	Troponin I 4.35 ng/mL	Not mentioned	CMR demonstrated LVEF = 64%, epicardial edema	No follow-up mentioned
			Chest pain	ST-segment elevation	Not mentioned	Troponin I 5.41 ng/mL	Not mentioned	CMR demonstrated LVEF 54%, epicardial and mid wall edema	No follow-up mentioned
7	Case report	Habib et al. (2021) [28]	Presented with chest pain preceded by generalized body aches, fever, chills, and headache for one-day	Mild ST-segment elevation	Ejection fraction (EF = 57%).	Troponin T (troponin T = 1138 ng\L).	The patient was started on dual antiplatelets, therapeutic anticoagulation, and metoprolol. After ruling out coronary artery disease at first, the received paracetamol IV for chest pain.	CMR revealed an early and late faint subepicardial enhancement of the basal lateral	Recovered and Discharged
8	Case series	Abbate et al. (2021) [34]	Presented in cardiogenic shock	ST-segment elevations	LVEF 20%	CRP(13.1 mg/dL),	Immunosuppressive Therapy Methylprednisolone 1000 mg	Not mentioned	died due to recurrent cardiac arrest and refractory shock
			Fever, cough, chest pain, nausea, and vomiting, hypotension and tachycardia	Not significant	LVEF of 15%	CRP 5.6 mg/dL	Immunosuppressive Therapy: Methylprednisolone	LVEF of 35%, small pericardial effusion, delayed enhancement after gadolinium at CMR	Recovered and discharged from the hospital after 73 days
9	Case series	Mouch et al. (2021) [19]	Chest pain	Diffuse ST elevation, Inverted T lead III	Normal	CRP - 58.1 mg/L; Troponin T - 589 ng/L;	NSAID and colchicine	T2 showed mild myocardial edema of the basal septum and inferolateral wall. Subepicardial and mid myocardial LGE of the same affected segments	Recovered and Discharged
			Chest discomfort	ST elevation V2-6, sinus tachycardia	LVEF of 50–55%	CRP level was 100.0 mg/L, Troponin T - 1062 ng/L.	Ibuprofen and colchicine	T2 sequence showed mild myocardial edema with LGE in the subepicardial region of the basal and middle anterolateral and inferolateral walls	Recovered and Discharged
			Chest pain	Diffuse ST elevation, Diffuse PR depression	Normal study	CRP - 86.0 mg/L, Troponin T - 876 ng/L	NSAID and colchicine	T2 sequences showed mild diffuse myocardial edema and LGE of the basal, inferolateral, anterolateral and anteroseptal walls	Recovered and discharged
			Chest pain	ST elevation: I, aVL, V3-5Inverted T, ST depression:III, aVF	LVEF- 50–55%.	CRP - 56.2 mg/L, Troponin T - 392 ng/L	Ibuprofen and Colchicine	LVEF 50–55%, T2 sequence showed subepicardial edema of the middle anterolateral, inferolateral and of the apical anterior walls with LGE of the affected walls	Recovered and discharged
			Chest pain	ST elevation V2-4	Normal	CRP -1.6 mg/L, troponin-I 14350 ng/L	Ibuprofen and Colchicine	LVEF 59%, mid myocardial and subepicardial edema of the basal inferolateral and middle anterolateral segments. LGE present in the same segments	Recovered and Discharged
			Chest pain	ST elevation I II aVL, V2–6SI QIII TIII	Normal	CRP - 54.7 mg/L, Troponin T 1130 ng/L	Ibuprofen and Colchicine	T2 sequence showed subepicardial edema of the basal inferolateral, middle inferolateral and infero-septal and apical lateral, anterior and inferior walls. LGE present in the same segments and mid-myocardial enhancement of the middle inferolateral and anterolateral and apical anterior and lateral walls.	Recovered and Discharged
10	Case report	Ammirati et al.(2021) [45]	Chest pain	Minimal ST elevation on precordial leads with peaked T waves	Not mentioned	Troponin T 289 ng/L, and C-reactive protein 2.9 mg/L	NSAIDs	LVEF (63%), There was focal subepicardial-intramyocardial on LGE involving the basal and apical segments of the infero-lateral wall, colocalized with signs suggestive for edema on T2 weighted images	Recovered and Discharged
11	Case report	Cereda et al. (2021) [51]	Fever and cardiac sounding chest pain	Diffuse ST elevation with slightly widened QRS	Normal	Troponin I: 6.53 ng/mL, C-reactive protein: 2.4 mg/dL	Bisoprolol and ramipril (beta-blocker + ACEi)	Epicardial edema and nonischemic delayed enhancement	Recovered and Discharged
12	Case series	Chamling et al. (2021) [21]	Acute chest pain with radiation to her left shoulder	No ST elevation	NOT significant	C-reactive protein [mg/L]: <0.6. cardiac troponin-T (hsTrop-T): elevated	aspirin, β-Blocker, ACE inhibitor, statine were prescribed on admission	LV-EF [%]:67	Follow-up not mentioned
			Chest discomfort	ST-segment elevations II, III, aVF	NOT significant	C-reactive protein [mg/L]:<0.08 with elevated hsTrop-T	Not mentioned	LV-EF [%]: 57	No follow-up mentioned
			Chest pain	ST-segment elevations II, III, aVF	Normal	C-reactive protein [mg/L]:13.2 with elevated hsTrop-T	Not mentioned	LV-EF [%]: 61,	No follow-up mentioned
13	Case report	D'Angelo et al.(2021) [52]	Dyspnea, constrictive retrosternal pain, nausea, and profuse sweating	Subtle ST-segment elevation	Preserved ejection fraction, mild pericardial effusion	Cardiac troponin I (12,564.80 pg/mL), C-reactive protein (39.6 mg/L).	Bisoprolol, acetylsalicylic acid, prednisolone.	LGE showed subepicardial enhancement of the myocardium	Recovered and Discharged
14	Case report	Deb et al. (2021) [18]	Dyspnea, fever, and chills, nausea, orthopnea, and increasing fatigue	Not significant	LVEF: 50%–54%	Troponin of 180.8 ng/L, CRP: 15.5 mg/Dl	Diuretics and supplemental oxygen therapy	NOT mentioned	Recovered and Discharged
15	Case series	Dickey et al. (2021) [30]	Positional and pleuritic chest pain, neck pain, chills and myalgias	Inferolateral ST elevation	Ejection fraction: 45%	Peak cardiac troponin I(ng/ml): 5.41	Not mentioned	CMR revealed patchy mid myocardial increased T2 signal with corresponding late gadolinium enhancement consistent with the acute inflammation of myocarditis	Recovered and Discharged
			Pleuritic and positional chest pain, rhinorrhea, headache and fever with 3 days into hospitalization.	Diffuse ST elevation	Ejection fraction: 53%	Peak cardiac troponin I(ng/ml): 38.3	Not mentioned	CMR revealed patchy midmyocardial increased T2 signal with corresponding late gadolinium enhancement consistent with the acute inflammation of myocarditis	Recovered and Discharged
			Pleuritic and positional chest pain, chills, myalgias and subjective fever	Sinus rhythm with diffuse ST elevation	Ejection fraction: 58%	Peak cardiac troponin I(ng/ml): 18.94	Not mentioned	CMR revealed patchy midmyocardial increased T2 signal with corresponding LGE consistent with the acute inflammation of myocarditis	Recovered and Discharged
			Chest pain radiating to back, myalgia, malaise and fever	Sinus rhythm with diffuse ST elevation	Ejection fraction: 48%	Peak cardiac troponin I(ng/ml):13.4	Not mentioned	CMR revealed patchy midmyocardial increased T2 signal with corresponding LGE consistent with the acute inflammation of myocarditis	Recovered and Discharged
			Pleuritic and positional chest pain, headache	NOT significant	Ejection fraction: 46%	Peak cardiac troponin I(ng/ml):5.21	Not mentioned	CMR revealed patchy midmyocardial increased T2 signal with corresponding LGE consistent with the acute inflammation of myocarditis	Recovered and Discharged
			Non-positional chest pain and myalgias	Ectopic atrial rhythm with diffude ST elevation	Ejection fraction: 50%	Peak cardiac troponin I(ng/ml):19.7	Not mentioned	CMR revealed patchy midmyocardial increased T2 signal with corresponding LGE consistent with the acute inflammation of myocarditis	Recovered and Discharged
16	Case report	Ehrlich et al. (2021) [36]	Fever, headache, chest pain and dyspnea.	Sinus rhythm	Ejection fraction: 45%	Troponin T concentration of 952 ng/L, elevated C-reactive protein (50.9 mg/L)	Therapy with acetylsalicylic acid, unfractionated heparin, an ACE inhibitor, a beta-blocker, and a mineralocorticoid antagonist was started.	Cardiac MRI revealed increased left ventricular wall thickness with a septal thickness of 16 mm at maximum and a persistent myocardial inflammation throughout the left ventricle: myocardial hyper-intensities on T2w images indicating myocardial edema were detected in the left ventricle, primarily in the basal and mid inferoseptal and anterolateral segments as well as in the apical lateral segment	Recovered and Discharged
17	Case report	Hasnie et al. (2021) [39]	Sharp substernal non-radiating chest pain, generalized body aches, and a subjective fever	Diffuse ST elevation.	LVEF: 50–55%	High sensitivity troponin was 13,702 ng/L	Aspirin, colchicine, metoprolol	Normal LVEF (58%), Mild adjacent pericardial LGE	Second dose administered with a course of NSAIDs
18	Case series	Hudson et al. (2021) [33]	Worsening myalgias and fevers, chills, nausea, vomiting, and 24 h of worsening midline, substernal burning that was worse when lying flat.	J‐point elevation in lateral leads with widened QRS complex	Normal	Troponin:1.5 ng/mL (<0.09), C‐reactive protein: 3.6 mg/dL	Aspirin and colchicine	CMR Not mentioned.	Recovered and Discharged
			presented to the ED with 3 days of worsening chills, low‐grade fevers, and chest pain	Normal ECG	Normal	Troponin:1.05 ng/mL (<0.09), C‐reactive protein: 3.6 mg/dL (<0.9 mg/dL).	Aspirin and colchicine, and ibuprofen	CMR not mentioned.	Recovered and Discharged
19	Case series	Larson et al. (2021) [17]	Fever, chills, mylagia on day +1, followed by chest pain day +3	Diffuse ST segment elevation with depression in aVR	LVEF: 50%,	Peak Troponin: 285 ng/L, CRP:4.8 mg/dL	NSAIDs, prednisone	Patchy subepicardial delayed enhancements	Recovered and Discharged
			Fever, chills, mylagia on day +1, chest pain, dyspnea on day +3	Normal ECG	LVEF: 34%, generalized hypokinesis	Peak Troponin: 46 ng/L, CRP: 14 mg/dL	Not mentioned	Patchy subepicardial and midmyocardial delayed enhancements	Recovered and Discharged
			Chest pain	Diffuse ST segment elevation	LVEF: 47%,	Peak Troponin: 520 ng/L, CRP: 9.5 mg/dL	Prednisone, colchicine	Edema, delayed enhancement, pericardial effusion	Recovered and Discharged
			Presented with chest pain	Diffused peak T wave	LVEF: 60%, inferiorlateral hypokinesis	Peak Troponin: 37 ng/L, CRP: 5.8 mg/dL	Not mentioned	Edema, delayed enhancement	Hemodynamically stable
			Cough, fever on day+1 and chest pain on day +3	Inferolateral ST elevation	LVEF: 60%, inferior wall hypokinesis	Peak Troponin: 100 ng/L, CRP: 1 mg/dL	Colchicine	Edema, delayed enhancement, pericardial effusion	Non sustained ventricular tachycardia(NSVT) episodes; discharged stable
			Fever on day +1 and chest pain on day +2	Diffuse ST segment elevation	LVEF: 50%, lateral and inferolateral hypokinesis	Peak Troponin: 29 ng/L, CRP: 9 mg/dL	NSAIDs	Edema, delayed enhancement	NSVT episodes; discharged stable
			Fever on day +1 and chest pain on day +4	Diffuse ST segment elevation	LVEF: 54%, inferior and posterolateral hypokinesis	Peak Troponin: 1164 ng/L, CRP: 4.6 mg/dL	NSAIDs	Edema, delayed enhancement, pericardial effusion and pericardial edema	NSVT episodes; discharged stable
			Chest pain on day 2	Inferolateral ST elevation	LVEF: 53%, in inferolateral hypokinesis	Peak Troponin: 1433 ng/L, CRP: 4 mg/dL	Not mentioned	Edema, delayed enhancement	NSVT episodes(N = 3); discharged table
20	Case Report	Khogali et al. . 2021 [46]	High-grade fever, fatigue, myalgia and headache. multi-organ failure, deranged liver function and DIC	Diffuse ST elevation and short PR interval	Ejection fraction (EF) of 27% increase in pericardial effusion, and signs of impending cardiac tamponade.	Troponin T increased from 98 ng/L reaching up to 1632 ng/L, CRP = 53.7 mg/L	Dobutamine, colchicine, and aspirin	CMR not mentioned	Admitted to ICU due to hemodynamic instability and the presence of combined hypovolemic, obstructive and cardiogenic shock. However recovered after 3 weeks and was discharged.
21	Case Report	Kim et al. . 2021 [16]	Chest pain that was not related with effort or labor; an atypical dull nature on the substernal area, and non-radiating and constant discomfort. Myalgias, fatigue	Mild ST-segment elevation in leads I, II,aVF, and V2-6	Minimal pericardial effusion. GLS bulls map revealed the worsened strain value in basal inferior and inferolateral segments, particularly in epicardium than endocardium.	Troponin-I 2.28 ng/mL, C-reactive protein 7.7 mg/dL	Symptomatic therapy	Abnormal findings on CMR, the subepicardial pattern of LGE in basal inferior and inferolateral segment	Recovered and discharged
22	Case Series	King et al. . 2021 [23]	Chest pain	Diffuse ST elevation and downsloping PR depressions	LVEF = 55–60%, with basal inferior and basal inferolateral hypokinesis	Troponin of 14,045 pg/mL and an elevated CRP.	Specific Treatment not mentioned	CMR revealed LGE involving the basal inferior, basal to mid inferolateral, mid anterolateral, apical lateral, apical septal, and apical inferior wall segments in a subepicardial distribution pattern, consistent with myocarditis.	Discharged on 3rd day of hospitalization
			Viral prodrome followed by chest pain	Diffuse ST elevations; downsloping PR depressions	LVEF of 45%moderate hypokinesis of the apex and apical septum.	Troponin-I was 22,638 and CRP was markedly elevated	Specific treatment not mentioned	Outpatient CMR is pending	Chest pain resolved the following day. He was discharged on hospital day 3.
			Chest pain	Diffuse ST elevations with no PR depressions	EF = 55%	Initial troponin-I was 3785 pg/mL and CRP was notably elevated.	Specific treatment not mentioned	An autoimmune workup showed an anti-nuclear antibody titer of 1:80 in a speckled pattern and negative double stranded DNA, rheumatoid factor, ribonucleic protein IgG, scleroderma-70, anti–Sjögren's-syndrome-related antigen A, and anti-Smith autoantibodies were negative	Discharged the following day
			Chest pain	T-wave inversions in the lateral leads that resolved on follow up ECG	Normal	Troponin-I was 2447 pg/mL and CRP was notably elevated.	Specific treatment not mentioned		Stabilized on same day and discharged on 3rd day
23	Case series	Koizumi et al. . 2021 [35]	Worsening chest pain	ST-elevation leads II, III, aVF and V3–6	Based on ECG and Labs findings	High-sensitivity troponin T (hsTnT) (0.906 ng/mL).	(NSAID) administration (ibuprofen 600 mg/day	Endomyocardial biopsy showed no inflammatory cell infiltration	Recovered and Discharged
			Worsening chest pain	Slight ST elevation	NOT significant	hsTnT (0.290 ng/mL	NSAID administration (ibuprofen 600 mg/day)	Cardiac MRI demonstrated LGE in the epicardial to the mid-wall in the left ventricle inferolateral wall	Recovered and Discharged
24	Case series	Mansour et al. . 2021 [15]	Fever and chills, Six hours later, developed substernal chest pain	Mild concave ST elevations	LVEF = 55%	Elevated troponin I of 14 ng/mL,(CRP) of 25 ng/mL (normal 0–0.5 ng/mL), CRP 25 ng/mL, ESR 25 mm/h.	Specific treatment not mentioned	Subepicardial LGE in the anterolateral wall of the mid and apical left ventricle.	Chest pain resolved. Patient discharged on day 3
			Light headedness. Two days later, developed retrosternal chest pain	Diffuse, mild concave ST elevations and PR depressions without reciprocal changes	LVEF = 50%	Elevated troponin I of 2.3 ng/mL, CRP of 8 ng/mL.	Specific treatment not mentioned	Subepicardial enhancement in the inferolateral wall at the base.	The patient's symptoms resolved the next day and her troponin declined to 1.3 ng/mL. The patient improved clinically and was discharged home on metoprolol.
25	Case Report	Matta et al. . 2021 [42]	Sharp, central, non-radiating chest pain associated with fatigue	Normal sinus rhythm without any ST-T changes.	EF = 60%	Elevated troponin I (0.245 ng/mL) and C-reactive protein (44.2 mg/L).	Aspirin 325 mg oral once	CMR not mentioned.	Patient stablilized and discharged the next day
26	Case Report	Muthukumar et al. . 2021 [27]	High fevers, shaking chills, myalgias, and a headache.	Sinus rhythm with left axis deviation and incomplete right bundle-branch block without ST or T wave changes	LVEF = 54%	Troponin I peaked at 6770 ng/L, C-reactive protein elevated	Low-dose lisinopril and carvedilol,	Midmyocardial and subepicardial linear and nodular LGE in the inferoseptal, inferolateral, anterolateral, and apical walls	At the time of discharge, the patient remained asymptomatic, and his high-sensitivity cardiac troponin T levels had fallen to 138 ng/L. His NT-proBNP (N-terminal pro-B-type natriuretic peptide) at discharge was <27 pg/mL.
27	Case Report	Nassar et al. . 2021 [26]	Developed dyspnea	Sinus tachycardia with a heart rate of 125bpm and T-wave inversions in leads V4–V6 without any ST-segment change	2+ aortic regurgitation and diffuse left ventricular hypokinesis with severely decreased LVEF = 10%	Troponin [1.260–2.050 ng/mL],	Multiple vasopressors + Antibiotics	The diagnostic monitoring via Swan-Ganz catheter revealed a pulmonary wedge pressure (PWP) of 14 mmHg	The patient declined cardiac catheterization and remained on medical therapy until her death on the eighth day of admission.
28	Case Series	Nevet et al. . 2021 [38]	Acute fever and chest pain	Diffuse ST elevations	Normal	Elevated inflammatory markers and myocardial enzymes	Colchicine and Ibuprofen	Edema and gadolinium enhancement of the myocardium were evident in cardiac magnetic resonance imaging, confirming the diagnosis of myocarditis	rapid clinical and laboratory improvement.
29	Case series	Patel et al., 2021 [31]	Chest pain, Headache, generalized malaise	Diffuse PR segment depression and PR segment elevation in lead aVR	LVEF = 55%	Serum TnI (ng/ml) = 37 C-reactive protein (mg/l) = 50	Aspirin and colchicine	Subepicardial LGE and myocardial edema in the basal inferior, basal inferolateral, and apical lateral LV segments.	discharged home in stable clinical condition after 48 h of observation
			Chest pain, Dyspnea, Nausea, emesis	Sinus tachycardia without any ST-T abnormalities	LVEF = 62% a	Serum TnI (ng/ml) = 49 C-reactive protein (mg/l) = 109	Colchicine and high-dose ibuprofen	CMR showed subepicardial LGE in the basal inferolateral segment. Diagnosed with myopericarditis,	Discharged in stable condition
			Chest pain, Dyspnea, Generalized body aches, nausea, headache, chills, and fatigue	Diffuse PR segment depression and PR segment elevation in lead aVR, consistent with acute pericarditis	LVEF = 60%	Serum TnI (ng/ml) = 17 C-reactive protein (mg/l) = 96	Colchicine	Subepicardial LGE and myocardial edema.)	Discharged in stable condition
			Chest pain, Fever, diaphoresis, rigors, nausea, myalgia, headache, fatigue	ST elevations in the lateral leads and ST depression in lead V1.	LVEF = 65%	Serum TnI (ng/ml) = 26 ESR 32 mm/h	None	CMR showed subepicardial LGE, myocardial edema.	Patient was home without any medications with cardiology follow up.
			Chest pain and dyspnea	PR segment depression and PR segment elevation	LVEF = 51%	Troponin I = 58 pg/mL	Colchicine, ibuprofen, lisinopril, and metoprolol tartrate	CMR showed subepicardial and mid-myocardial LGE in the basal, mid, and apical lateral segments accompanied by myocardial edema in mid and apical lateral segments on T2-weighted images	Recovered and Discharged
30	Case series	Rosner et al., 2021 [2]	Chest pain at rest, nonpleuritic, non- exertional; no fevers, cough- ing, or dyspnea	ST elevation	LVEF = 51%, mid global hypokinesis	Cardiac trop I 17.08 ng/mL	Beta blockers, ACE inhibitors	Patchy mid subepicardial LGE	Recovered and Discharged
			Chest pain associ- ated with dyspnea; worse when lying flat and with inspiration	PR depression in II, aVF, V4–V6, T wave inversion V1	LVEF = 35–40%	Cardiac troponin I ng/mL peak = 11.01, C-reactive protein peak, mg/dL = 1.3	β-blocker, angioten- sin-converting en- zyme inhibitor, aspi- rin, and clopidogrel	Patchy mild sub- epicardial LGE, No definitive edema	Recovered and Discharged
			Fever, chills, dyspnea, and chest heaviness/pain symptoms	Not significant	LVEF = 61%	Cardiac troponin I ng/mL peak = 13, C-reactive protein peak, mg/dL = 5.1	β-blocker, angio- tensin receptor blocker, statin	Left ventricular ejection fraction = 56% (no region- al wall motion abnormalities) LGE = Subepicardial LGE, no pericardial thickening or ef- fusion	Recovered and Discharged
			Intermittent, positional chest pain with left arm numbness and tingling	Not signficant	LVEF = 53%	Cardiac troponin I ng/mL peak = 0.37, C-reactive protein peak, mg/dL = 11.70	3 days IV steroids	Left ventricular ejection fraction = 52%, Multifocal sub- epicardial and midmyocardial LGE	Recovered and Discharged
			Midsternal sharp chest pain, waxing/and po- sitional; relieved with leaning forward	Not significant	LVEF = 55%	Cardiac troponin I ng/mL peak = 44.8, C-reactive protein peak, mg/dL = 0.1	Colchicine, ibuprofen, fa- motidine	Left ventricular ejection fraction = 48% Midmyocardial LGE; no pericardial effusion T2 = Myocardial edema	Recovered and Discharged
			Midsternal chest pain with deep inspiration	ST elevation in V2-5	LVEF = 50–55%	Cardiac troponin I ng/mL peak = 8.36 ng/mL in patient 6 and cTnI 2601 ng/L in patient 7	Ibuprofen, famotidine	Left ventricular ejection fraction = 50%,Multifocal patchy subepi- cardial and mid- myocardial LGE T2 = Myocardialedema in patient 6 and basal midwall anteroseptal delayed enhancement in patient 7	Recovered and Discharged
			Fevers, diffuse myalgias, and headache starting day of vaccination; sudden onset of sharp chest pain the night before admission that persisted at 3 out of 10 intensity, worsened when lying flat	Diffuse ST elevation	LVEF = 58%,	High sensitive Cardiac trop I 2601 ng/mL	Beta blockers, colchicine	Patchy mid subepicardial LGE	Recovered and Discharged
31	Case report	Singh et al., 2021 [40]	Chest pain:left-sided, severe, constant, non-radiating, was associated with headache	ST-depression in lead III	LVEF = 55%	Cardiac troponin I ng/mL peak = 8.36, C-reactive protein peak, mg/dL = 8.2	No specific treatment mentioned	Left ventricular ejection fraction = 52%, Subepicardial LGE T2 = inferior wall myocardial edema	The patient was hospitalized for 4 days and discharged in a stable condition. He was seen in an outpatient clinic 6 weeks later, is doing well and is back at work.
32	Case report	Sokolska et al., 2021 [20]	Severe chest pain	Q wave and ST-segment elevation in leads II, III and aVF	LVEF = 58%	high-sensitive troponin (6490–6559 pg/mL; reference range <34 pg/mL), C-reactive protein (82 mg/L; reference range <5 mg/L)	Not mentioned	Diffuse subepicardial LGE	Recovered and Discharged
33	Case series	Starekova et al., 2021 [25]	Chills, headache, fever, chest discomfort and pain, dyspnea	Diffuse ST-elevations	LVEF = 32%	troponin (6490–6559 pg/mL), C-reactive protein (82 mg/L).	Not mentioned	Linear, midmyocardial septum, epicardial LGE	Follow-up not mentioned
			Headache, body ache, fatigue, chest discomfort and pain	Nonspecific T-wave abnormality	LVEF = 64%	Troponin-I at peak (ng/mL) = 3.82	Not mentioned	Pericardial enhancement and small effusion. Epicardial LV LGE	Follow-up not mentioned
			Subjective Mild Fever, Chills, Malaise,Nausea, Chest pain	diffuse ST elevations	LVEF = 53%	Troponin-I at peak (ng/mL) = 1.02BNP (pg/mL) = 75	Not mentioned	LGE epicardial LV, Pericardial enhancement, no effusion.	Follow-up not mentioned
			Subjective Mild Fever, Chills, Malaise,Nausea, Chest pain	Nonspecific T-wave abnormality	LVEF = 57%	Troponin-I at peak (ng/mL) = 14.65	Not mentioned	Epicardial LV, Pericardial enhancement, no effusion. Epicardial LV LGE	Follow-up not mentioned
			Myalgias, Malaise, Nausea, lightheadedness, chest pain	Inferolateral T-wave inversion	LVEF = 54%	Troponin-I at peak (ng/mL) = 4	Not mentioned	LGE: epicardial LV. Pericardial enhancement and borderline effusion.,	Follow-up not mentioned
34	Case report	Tailor et al., 2021 [47]	Severe chest pain radiating to both arms, associated with acute dyspnea	ST segment elevation in lateral limb and precordial leads	LVEF = 40% with global hypokinesia mainly at apex	Troponin-I at peak (ng/mL) = 12.19	Brief course of intravenous diuretics for mild symptoms of congestion. Angiotensin-converting enzyme inhibitor and a beta-blocker therapy were commenced to treat systolic dysfunction. He was also initiated on colchicine to treat mild persistent chest pain	patchy linear mid-myocardial enhancement of the septum and inferior walls at the base to mid-ventricle, sub-epicardial/mid-myocardial enhancement of the lateral wall at the mid-ventricle and apical lateral wall	He was discharged home after 5 days of monitoring without evidence of electrical or haemodynamic instability and with NYHA Class I symptoms
35	Case report	Ujueta et al., 2021 [37]	Progressive body aches, weakness and worsening fatigue	Sinus tachycardia with T wave inversions in the septal leads with right atrial enlargement	Biventricular cardiomyopathy with LVEF = 29%, and a small pericardial effusion	C-reactive protein 63.5 <_8.0 mg/L Peak troponin T, 847	Vasopressin, Phenylephrine, and Epinephrine. Intravenous (IV) Methylprednisolone 60 mg bolus was administered every 8 h,	Multiple immunohistochemistry staining like CD163 supports the diagnosis of lymphohistiocytic myocarditis with sparse eosinophils	patient expired after several rounds of advanced cardiovascular life support. Consent was obtained for an autopsy.
36	Case series	Verma et al., 2021 [41]	Dyspnea and dizziness	Tachycardia; STsegment depression	Severe biventricular cardiomyopathy with LVEF = 29%, and a small pericardial effusion	Cardiac Marker Troponin I 6.4 ng/mL peak, C-Reactive protein = 49	Vaso-pressin, Phenylephrine, and Epinephrine. Intravenous (IV) Methyl-prednisolone	CMR Not mentioned, endomyocardial biopsy confirmed the diagnosis	Recovered and Discharged
			Dyspnea and chest pain	Diffuse ST-segment elevation	LVEF = 15%	Troponin I level of 6.14 ng per milliliter	Inotropic support, intravenous diuretics, methylprednisolone and guidelinedirected medical therapy for heart failure (lisinopril, spironolactone, and metoprolol succinate)	Histologically confirmed during autopsy	Patient expired
37	Case report	*Williams* et al., 2021 [50]	Fevers and myalgias and dull, retrosternal chest pain	Lateral PR depression and ST elevation mirrored in aVR with PR elevation and ST depression	LVEF = 43%	Troponin T 1.30 ng/mL; CRP 10.2 mg/dL	Ibuprofen	Slightly reduced left ventricular pump function, myocardial edema, and subepicardial late gadolinium enhancement	Recovered and Discharged
38	Case series	Levin et al.(2021) [24]	Fatigue, headache, abdominal pain, chest pain radiating to right arm, perspiration	ST elevation	LVEF = 43%	Troponin T (hs-cTnT) concentration of 4026 ng/L, and C-reactive protein 111 mg/L	High dose aspirin, colchicine, bisoprolol and rampiril	LVEF to 54% with subepicardial late gadolinium enhancement, patchy myocardial edema, pericardial enhancement	Recovered and Discharged
			Abdominal pain, chest and fatigue	ST elevation, inferiorleads, reciprocal depression on leads I and AVL.	LVEF = 45%	Troponin-I − 22,000 ng/L (0–50),CRP- 58.54 mg/L (<0.03–5)	Bisoprol. Ramipril	Cardiac spectral CT-sub epicardial focal enhancement of the lateral wall and septum of the inferior wall, Nopericardial or pleural effusion	Recovered and Discharged
			Fatigue, throat pain and dizziness	Normal ECG	LVEF = 60%	Troponin-I15,000 ng/dL (0–50), CRP9 mg/L (<0.03–5)	Bisoprol, Ramipril	Cardiac CT- late adherence through the lateral wall, the inferior basal wall, the apex and middle part of theseptum	Recovered and Discharged
			Chest pain radiating to the left arm, fatigue	ST-elevation I, II, III,AVF, V3-6 leads	LVEF = 60%	Troponin-I15,527 ng/dL (0–50),CRP- 44 mg/L (<0.03–5)	Bisoprol, Ramipril	Cardiac MRI(two weeks after discharge) -LV EF51%, and late subepicardial and mesocardiac enhancement of 5% of LV wall	Recovered and discharged
			Squeezing chest pain and dyspnea	Diffuse ST-segment elevation in septal and lateral leads, and PR segment depressions in inferior leads.	Normal	Troponin-I6000 ng/Dl, CRP7 mg/L (0.2–5)	Colchicine, Ibuprofen	Not mentioned	Recovered and Discharged
			Fever and malaise, squeezing chest pain radiating to the back	Sinus tachycardia and findings consistent with LV hypertrophy.	LVEF = 60%	Troponin-T409 ng, CRP = 58.1 mg/L (0.2–5)	Colchicine, Ibuprofen	Myocardial edema and LGE were noted in the basal LV and subepicardial myocardium	Recovered and Discharged
			Stabbing chest pain aggravated by lying down, myalgia, headache, malaise	Diffuse ST-segment elevation associated with ST-segment depression in AVR and PR segment depression.	Normal	**Troponin-T** – 33 ng/L (0–14), **CRP** − 4 mg/L	Colchicine	Not mentioned	Recovered and Discharged
39	Case report	Patrignani et al. (2021) [22]	Stabbing chest pain aggravated by lying down, myalgia, headache, malaise	Diffuse ST-segment elevation associated with ST-segment depression in AVR and PR segmentedession	LVEF = 38–40%	Troponin-T-2300 ng/L (0–20), CRP = 120 mg/L (0–5).	Colchicine, Bisoprol	LGE in subepicardial and midmyocardium along the lateral wall, infero-basalwall and mid- and basal septum involving 8% of myocardial mass.	Recovered and Discharged
40	Case report	Sulemankhil et al.(2021) [49]	Acute substernal chest pain followed by constant, retrosternal, non-radiating, non-exertional chest pain.	Normal ECG	Normal	Troponin T 0.041 ng/mL (Normal range <0.014 ng/mL), CRP = 40.4 mg/L	Not mentioned	A gadolinium-enhanced cardiac magnetic resonance imaging showed a small focal area of myocarditis in the mid to apical lateral region of the left ventricle with a scar size of 2%	Recovered and Discharged
41	Case report	Garcia et al. (2021) [13]	Fever. Intermittent chest and interscapular pain	Diffuse ST-segment elevation	NOT significant	Troponin T (hsTnT) of 139 ng/L.	Anti-inflammatory treatment	Subepicardial edema	Follow-up not mentioned
LGE: Late Gadolinium Enhancement LVEF: Left Ventricular Ejection Fraction, EF: Ejection Fraction, CRP: C-Reactive Protein, TTE: transthoracic Echocardiogram, CMR: Cardiac Magnetic Resonance Imaging, ECG: Electrocardiogram, LV: Left Ventricle.									

Original Article:												
Sr No	Study Design	Author, Year	Country reported	Sample size	Age	Gender (M/F)	Follow-up	Comparator	Experimental group characteristics (Vaccine administered)	Outcome		Results
											Clinical features	
1	Retrospective observational study	George A. Diaz, 2021 [54]	USA	2000287	median = 57(40–70)	822118/1178169	N/A	None	BNT162b2(Pfizer/BionTech) = 52.6%, mRNA-1273(Moderna) = 44.1%, Ad26.COV2.S(Janssen/Johnson & Johnson) = 3.1%, 76.1% received more than 1 dose	Myocarditis = 20, pericarditis = 37 where 4 participants developed myocarditis after first dose and 16 developed after second dose, 15 participants developed pericarditis after first dose and 22 developed after second dose	Mild Myocarditis = 20 (100%)	Rate Ratio: myocarditis: (1.0 [95% CI, 0.61–1.54] per 100,000), Pericarditis:(1.8 [95% CI, 1.30–2.55] per 100,000)
											Mild Pericarditis = 37 (100%)	
											ECG changes = 23 (40%)	
											Abnormal EF = 8 (14%)	
											Elevated Troponin = 9 (16%)	
2	Prospective cohort study	Han. W. Kim, 2021 [56]	USA	4	Mean:38.5	3/1	N/A	None	BNT162b2(Pfizer/BionTech) = 2, Moderna (mRNA1273) = 2, Dose = 2, Onset of symptoms = 3 days after vaccination for patient 1,5 days after vaccination for patient 2, 1 day after vaccination for patient 3, 2 days after vaccination for patient 4	Myocarditis in all four patients	Mild myocarditis = 4 (100%)	N/A
											Chest pain = 4 (100%)	
											Dyspnea = 3 (75%)	
											Fever = 3 (75%)	
											ECG changes = 4 (100%)	
											ST-segment elevation = 4 (100%)	
											Elevated Troponin = 4 (100%)	
											C-reactive protein = 3 (75%)	
											LGE = 4 (100%)	
											LVEF (abnormal) = 1 (25%)	
3	Retrospective cohort study	Guy Witberg, 2021 [57]	Israel	2,558,421	median44 [[30], [31], [32], [33], [34], [35], [36], [37], [38], [39], [40], [41], [42], [43], [44], [45], [46], [47], [48], [49], [50], [51], [52], [53], [54], [55], [56], [57], [58], [59], [60], [61], [62], [63]]	1,248,433/1,309,988	42 days	None	BNT162b2(Pfizer/BionTech) = 2,558,421, All of the participants received first dose. whereas 2,401,605 participants received second dose.	Myocarditis = 54	Mild myocarditis = 41(76%) Intermediate myocarditis = 12 (22%). LVD = 14(26%)	Cumultative incidence: 2.13(1.56–2.70)per 1,00,000
											chest pain = 44 (81%)	
											Dyspnea = 3 (5.5%)	
											Fever = 5 (9%)	
											Pericardial effusion = 10 (18.5%)	
											ECG changes = 38 (70%)	
											Elevated Troponin T = 41	
4	Retrospective cohort study	Noam Barda, 2021 [53]	USA	1,736,832	median age 38	903153/833679	42 days	Unvaccinated = 884828	Vaccinated group,BNT162b2(Pfizer/BionTech):884,828	In vaccinated group: Myocarditis:21 Pericarditis:27 In non-vaccinated group: Myocarditis: 6, pericarditis: 18	N/A	Risk Ratio: Myocarditis: 3.24 (1.55–12.44), Pericarditis:1.27 (0.68–2.31)
5	Retrospective cohort study	Mevorach et al., 2021 [55]	Israel	9,289,765	–	2,668,894/2,773,802	183 days	None	BNT162b2(Pfizer/BionTech) = 5,442,696. 1st dose: 5,442,696, 2nd Dose: 5,125,635	Myocarditis: 136, After first dose:19 After second dose: 117	Mild myocarditis: 129 (90.9%) cases.	RD (95%CI) for myocarditis according to age and sex (21 days after first dose): 3.19 (2.37–4.02) Standardized Incidence ratio for myocarditis according to age, sex and dose: 5.34 (4.48–6.40), Rate ratio of myocarditis within 30 days after second dose as compared to unvaccinated patients: 2.35 (1.10–5.02)
											Chest pain = 129 (95%)	
											Fever = 63 (46.7%)	
											Dyspnea = 17 (12.5%)	
											ECG changes = 93 (68%)	
											Elevated Troponin I or T = 136 (100%)	
											Elevated C-reactive protein = 118 (86.7%)	
											LGE = 48 (35%)	

LVEF = Left Ventricular Ejection Fraction, EF = Ejection Fraction, LGE = Late Gadolinium enhancement, LVD = Left Ventricular Dysfunction, ECG = Electrocardiogram, RD = Risk difference.

The mean age of patients was 29.34 ± 12.94 years (range 16–68). The highest number of cases were reported in the USA (n = 23, 56.09%). The majority of the cases were seen in males (n = 83, 85.5%). Only 10 patients (10.3%) had a positive history of SARS-CoV-2 infection and 6 (6.1%) had a history of some cardiovascular disease. Out of the 97, most of the patients received Pfizer-BioNTech (n = 67, 69%) and rest of the patients received 25 (25.7%) Moderna (n = 67, 69%), Janssen Johnson & Johnson (n = 4, 4.1%) and AstraZeneca (n = 1, 1.03%). A total of 79 (81.4%) patients developed acute myocarditis, 9 (9.2%) myopericarditis or perimyocarditis, 3 (3%) acute pericarditis, 4 (4.1%) fulminant myocarditis, 1 (1.03%) each with fulminant pericarditis and lymphohistiocytic myocarditis. The majority of the patients developed the symptoms after the second dose of the vaccine (n = 77, 79%). Chest pain (n = 88, 90%), fever (n = 33, 34%), dyspnea (n = 18, 18.5%), and myalgias (n = 18, 18.5%) were the most common presentations. The mean time between the administration of the vaccine and the development of symptoms was 3.8 ± 4.53 days.

On investigations, 62 (63.9%) patients had ST-segment elevation, 12 (12.3%) had normal ECG and ECG changes of 5 (5.1%) patients were not mentioned. Echocardiogram findings demonstrated that 65 (67%) patients had preserved ejection fraction, 27 (27.8%) had decreased ventricular ejection fraction and echocardiogram findings were not mentioned for 5 (5.1%). Most of the patients (n = 88, 90.7%) had elevated levels of serum cardiac troponin while almost half (n = 55, 56.7%) also had elevated levels of C-reactive protein. CMR findings were supportive for myocarditis or pericarditis in 84 (86.6%) patients. In 11(11.3%) patients, CMR was not performed, and 2 (2%) patients had their diagnosis confirmed by biopsy and Swan-Ganz catheterization, respectively. The management included colchicine (n = 29, 29.8%), beta-blockers (n = 22, 22.6%), aspirin (n = 11, 11.3%) and other anti-inflammatory drugs (n = 21, 21.6%). Out of 97, 80 (82.5%) patients recovered, 4 (4.1%) patients expired and follow-up was not mentioned for the remaining 13 (13.4%) patients.

### 3.2 Original articles

There were 15,585,309 participants included in five original articles. Three studies were conducted in USA (United States of America) and two in Israel. Out of 15,585,309 participants, 6,095,639 (39.11%) were females and 9,489,670 (60.8%) were males. A total of 9,938,097 (63.7%) participants received Pfizer/BioNTech, 882,128 (5.6%) received Moderna and 62,008 (0.4%) received Janssen/Johnson & Johnson. Out of these patients, 235 (0.001%) developed myocarditis and 64 (0.0004%) developed pericarditis. The mild cases of myocarditis among these were 194 (82%) whereas all 64 (100%) cases of pericarditis were described as mild. Majority of the patients presented with chest pain (n = 177, 75%), fever (n = 71, 30%) and dyspnea (n = 23, 10%). Investigations of these patients revealed raised troponin (n = 190, 80%), ECG changes (n = 158, 67%), Late Gadolinium Enhancement (LGE) (n = 48, 20%), left ventricular dysfunction (LVD) (n = 14, (6%) and abnormal EF (n = 8, 3.4%). All the participants received the first dose of the vaccine while 9,047,460 (58%) participants also received the second dose of the vaccine. The mean follow-up reported by three articles was 89 days.

## 4 Discussions

This systematic review summarized evidence from the original studies, case reports, and case series which discussed the development of myocarditis and pericarditis following COVID-19 vaccination. This will keep physicians up-to-date regarding the complications and side effects of newly introduced COVID-19 vaccines. We found that males are notably more likely to develop myocarditis and pericarditis following COVID-19 vaccination than females (85% vs 15%). The majority of the patients had no significant history of COVID-19 infection or any other cardiovascular disease. The prevalence of myocarditis and pericarditis was more among the patients who received Pfizer-BioNTech (BNT162b2) than those who received other vaccines, but this may be due to the fact that more patients included in this review had received the aforementioned vaccine. Similarly, a greater percentage of patients who developed the symptoms received two doses of vaccine (compared to one). Chest pain, fever, myalgias, and dyspnoea were the most common presentations. The majority of the patients who presented with myocarditis and pericarditis had a good recovery and were discharged.

Several hypotheses have been put forward to explain the factors that might cause these complications of the COVID-19 vaccine. However, the exact pathophysiology is yet to be elaborated. One of the proposed mechanisms is the interaction between components of the vaccine and the susceptibility of the subject known as molecular mimicry. Due to the similarity between the pathogenic component of the vaccine and specific human proteins, there is immune cross-reactivity resulting in autoimmune disease [58,59]. Among other vaccines for which myocarditis has been reported as an adverse effect, only the smallpox vaccine has demonstrated a significantly high risk [60]. However, the smallpox vaccine differs from the COVID vaccine both in composition and elicitation of a specific immune response.

The higher prevalence of this condition among males can be explained based on the role played by variations in hormone signalling. Testosterone has the ability to suppress anti-inflammatory immune cells while promoting a more aggressive T helper 1 cell immunological response. Oestrogen, on the other hand, inhibits pro-inflammatory T cells, resulting in a reduction in cell-mediated immune responses [59]. However, further research is required to explore the exact phenomenon.

The incidence of myocarditis following the second dose is greater, probably because of a phenomenon called hypersensitivity myocarditis, with the first dose presenting as a sensitising dose [61]. More prevalence of myocarditis and pericarditis among the patients who received Pfizer-BioNTech (BNT162b2) and Moderna (mRNA 1273) indicates that mRNA vaccines are associated with a higher risk of developing myocarditis than the viral vector vaccines like AstraZeneca and The Janssen/Johnson & Johnson [62]. Bozkurt et al. has proposed that autoantibody generation and subsequent attack on cardiac myocytes in response to mRNA vaccine underlie this increased risk [63]. Larger scale studies have indicated myocarditis and pericarditis to be rare adverse events of the COVID-19 vaccine. The US population-based study has reported the incidence rate of myocarditis and pericarditis to be 5.73 to 26 cases per 100,000 person-year and 0.95 to 2.16 cases per 100,000 person-year, respectively [64]. Another study conducted in Israel has reported the cumulative incidence rate to be 2.13 (1.56–2.70) per 100,000 [65].

Most patients underwent CMR imaging revealing myocardial edema and hyperaemia, findings supportive of myocarditis. CMR imaging has an important role in therapeutic decision-making in patients with suspected myocarditis. It acts as a predictor of functional and clinical recovery and the CMR-visualised pattern of myocardial damage provides some insight into the underlying illness aetiology and pathogenesis [66]. As the CMR imaging of patients was performed in an acute setting, it was difficult to assess the actual degree of damage and prognosis and highlight etiological and pathological factors that may be at play [67]. NSAIDs, colchicine, and steroids were the most commonly employed treatments in the case studies, suggesting that the management of post-COVID vaccine myocarditis is in line with the current guidelines. The good prognosis and recovery of patients in most cases corroborate this fact as well. The effectiveness of anti-inflammatory drugs also backs the theory of molecular mimicry and autoimmunity in C-VAM (COVID vaccine-associated myocarditis).

Practising physicians and healthcare providers can benefit from the information included in this study by providing improved consultation on vaccine safety and potential side effects. Healthcare providers should discuss all the possible risk factors before choosing the specific type of vaccine. The viral vector vaccine can be an alternative for patients with increased risk of myocarditis/pericarditis, or for those who have a history of cardiomyopathy …

The main limitation of this review is that no large-scale clinical trial investigating the risk factors, clinical presentation, and prognosis of patients developing myocarditis and pericarditis following COVID-19 vaccination has been conducted so far so only case reports, case series, and cohort studies have been included in the review. Moreover, there is inherent heterogeneity owing to the individual nature of every patient included in the case report and case series. Lastly, mild cases of myocarditis and pericarditis remain unreported and due to the recent nature of the condition, there is insufficient evidence to expound on the underlying pathogenic mechanisms. There is a significant potential for publication bias because rare events and diagnostically unique cases are more likely to be reported and published.

## 5 Conclusion

Myocarditis and pericarditis after the COVID-19 vaccine occur most commonly in adult males after the second dose of mRNA vaccines (Pfizer and Moderna). The presentation is usually mild, and the majority of patients have a good recovery. Cell-mediated immune responses generated by the body against the vaccine components cross-react with cardiac cells to cause myocardial and pericardial inflammation. It follows that the most effective treatment for this clinical entity are immunosuppressants and anti-inflammatory agents (e.g., colchicine, NSAIDs and steroids). Physicians should consider myocarditis and pericarditis as a probable diagnosis in patients who have received COVID-19 vaccines, especially in males who develop suggestive symptoms after a second dose of Pfizer and Moderna. Viral vector vaccines may be a better alternative for patients with a history of cardiac diseases.

## Ethical approval

This is a systematic review and did not require ethical approval.

## Sources of funding

This research did not receive any grant from funding agencies in the public, commercial, or not-for-profit sectors.

## Author contribution

MF and HAC conceived the idea established a search strategy. MF, MHAK and MSA retrieved the articles, and screened them for relevancy. After selecting relevant articles, MWM, UH and HS ran quality assessment on the included articles. Data was extracted by MF, UH, MHAK and HS. HF and MAUR proofread the extracted data and matched it with articles to eliminate errors. MF and MHAK then worked on the write up. MAUR, HF and HAC provided critical assistance in proof reading and editing of the write up. All the authors approved the final version of the article.

## Registration of research studies

Name of the registry: PROSPERO.

Unique Identifying number or registration ID: CRD42021276596.

Hyperlink to your specific registration: https://www.crd.york.ac.uk/PROSPERO/display_record.php?RecordID=276596.

## Guarantor

I, Maurish Fatima, the corresponding author for this review accept my role as the Guarantor for this research.

## Consent

This is a systematic review, where authors verified that proper consent was obtained from patients in all of the studies included.

## Provenance and peer review

Not commissioned, externally peer-reviewed.

## Declaration of competing interest

The authors declare no conflict of interest.
