# Supplementary material for: Development of myocarditis and pericarditis after COVID-19 vaccination in adult population: A systematic review
Source: Ann Med Surg (Lond). 2022 Mar 11;76:103486. doi: 10.1016/j.amsu.2022.103486 (PMC8912977; doi:10.1016/j.amsu.2022.103486)
Supplement: Multimedia component 1 [file mmc1.docx]

**Supplementary table_1:Scoring of Case Series on Critical Appraisal**

| Sr. No | Study Author, year | Clear criteria for inclusion | Condition measured in standard and reliable way | Usage of valid methods for identification of condition | Consecutive inclusion of participants | Clear reporting of demographics | Clear reporting of outcomes or follow ups | Clear reporting of presenting site/clinics demographic information | Appropriate statistical analysis | Score | Inclusion/Exclusion | Additional Comment |
| --- | --- | --- | --- | --- | --- | --- | --- | --- | --- | --- | --- | --- |
| 1 | Vidula et al, 2021 | 1 | 1 | 1 | 1 | 0 | 1 | 1 | 1 | 7 | Included | No clear reporting of demographics of patients |
| 2 | Shaw et al, 2021 | 1 | 1 | 1 | 1 | 0 | 1 | 1 | 0 | 6 | Included | No clear reporting of demographics of patients |
| 3 | Abbate et al (2021) | 1 | 1 | 1 | 1 | 1 | 1 | 1 | 0 | 7 | Included | Appropriate statistical analysis is not present |
| 4 | Mouch et al (2021) | 1 | 1 | 1 | 1 | 1 | 1 | 1 | 1 | 8 | Included | None |
| 5 | Chamling et al,2021 | 1 | 1 | 1 | 1 | 1 | 1 | 1 | 0 | 7 | Included | Appropriate statistical analysis is not present |
| 6 | Larson et al, 2021 | 1 | 1 | 1 | 1 | 1 | 1 | 1 | 1 | 8 | Included | None |
| 7 | Hudson et al,2021 | 1 | 1 | 1 | 1 | 1 | 1 | 1 | 0 | 7 | Included | Appropriate statistical analysis is not present |
| 8 | Dickey et al, 2021 | 1 | 1 | 1 | 1 | 1 | 1 | 1 | 0 | 7 | Included | Appropriate statistical analysis is not present |
| 9 | King et al, 2021 | 1 | 1 | 1 | 1 | 1 | 1 | 1 | 1 | 8 | Included | None |
| 10 | Koizumi et al, 2021 | 1 | 1 | 1 | 1 | 1 | 1 | 1 | 0 | 7 | Included | Appropriate statistical analysis is not present |
| 11 | Mansour et al, 2021 | 1 | 1 | 1 | 1 | 1 | 1 | 1 | 0 | 7 | Included | Appropriate statistical analysis is not present |
| 12 | Patel et al, 2021 | 1 | 1 | 1 | 1 | 1 | 1 | 1 | 1 | 8 | Included | None |
| 13 | Rosner et al, 2021 | 1 | 1 | 1 | 1 | 1 | 1 | 1 | 1 | 8 | Included | None |
| 14 | Starekova et al, 2021 | 1 | 1 | 1 | 1 | 1 | 1 | 1 | 0 | 7 | Included | Appropriate statistical analysis is not present |
| 15 | Verma et al, 2021 | 1 | 1 | 1 | 1 | 1 | 1 | 1 | 0 | 7 | Included | Appropriate statistical analysis is not present |
| 16 | Levin et al, 2021 | 1 | 1 | 1 | 1 | 1 | 1 | 1 | 1 | 8 | Included | None |

**Supplementary table_2:Scoring of Case Reports on Critical Appraisal**

| Sr No | Study Author, Year | Clear Description of Demographic characteristics | Clear Description of history and presentation as a timeline | Clear Description of current clinical condition of patient | Clear Description of diagnostic tests or assessment methods | Clear description of intervention or treatment procedure | Clear description of post-intervention clinical condition | Identification of Adverse events or unanticipated events | Take way lessons | Score | Inclusion/Exclusion | Additional Comments |
| --- | --- | --- | --- | --- | --- | --- | --- | --- | --- | --- | --- | --- |
| 1 | Cimaglia et al, 2021 | 1 | 1 | 1 | 1 | 0 | 0 | 1 | 1 | 6 | Included | Clear description of intervention is not present |
| 2 | Ngyuen et al, 2021 | 1 | 1 | 1 | 1 | 0 | 0 | 1 | 1 | 6 | Included | Clear description of intervention is not present |
| 3 | Watkins et al, 2021 | 1 | 1 | 1 | 1 | 1 | 1 | 1 | 1 | 8 | Included | None |
| 4 | Albert et al, 2021 | 1 | 1 | 1 | 1 | 1 | 1 | 1 | 1 | 8 | Included | None |
| 5 | Habib et al, 2021 | 1 | 1 | 1 | 1 | 1 | 1 | 1 | 1 | 8 | Included | None |
| 6 | Ammirati et al,2021 | 1 | 1 | 1 | 1 | 1 | 1 | 1 | 1 | 8 | Included | None |
| 7 | Cereda et al,2021 | 0 | 1 | 1 | 1 | 1 | 1 | 1 | 1 | 8 | Included | None |
| 8 | D'Angelo et al, 2021 | 1 | 1 | 1 | 1 | 1 | 1 | 1 | 1 | 8 | Included | None |
| 9 | Deb et al, 2021 | 1 | 1 | 1 | 1 | 1 | 1 | 1 | 1 | 8 | Included | None |
| 10 | Hasnie at al, 2021 | 1 | 1 | 1 | 1 | 1 | 1 | 1 | 1 | 8 | Included | None |
| 11 | Ehrlich *et al,* 2021 | 1 | 1 | 1 | 1 | 0 | 0 | 1 | 1 | 6 | Included | Clear description of intervention is not present |
| 12 | Khogali et al, 2021 | 1 | 1 | 1 | 1 | 1 | 1 | 1 | 1 | 8 | Included | None |
| 13 | Kim et al, 2021 | 1 | 1 | 1 | 1 | 1 | 1 | 1 | 1 | 8 | Included | None |
| 14 | Matta et al, 2021 | 1 | 1 | 1 | 1 | 1 | 1 | 1 | 1 | 8 | Included | None |
| 15 | Muthukum et al, 2021 | 1 | 1 | 1 | 1 | 1 | 1 | 1 | 1 | 8 | Included | None |
| 16 | Nassar et al, 2021 | 1 | 1 | 1 | 1 | 1 | 1 | 1 | 1 | 8 | Included | None |
| 17 | Nevet et al, 2021 | 1 | 1 | 1 | 1 | 1 | 1 | 1 | 1 | 8 | Included | None |
| 18 | Singh et al, 2021 | 1 | 1 | 1 | 1 | 1 | 1 | 1 | 1 | 8 | Included | None |
| 19 | Sokoloska et al, 2021 | 1 | 1 | 1 | 1 | 0 | 0 | 1 | 1 | 6 | Included | Clear description of intervention is not present |
| 20 | Tailor et al, 2021 | 1 | 1 | 1 | 1 | 1 | 1 | 1 | 1 | 8 | Included | None |
| 21 | Ujueta et al, 2021 | 1 | 1 | 1 | 1 | 1 | 1 | 1 | 1 | 8 | Included | None |
| 22 | Wlliams et al, 2021 | 1 | 1 | 1 | 1 | 1 | 1 | 1 | 1 | 8 | Included | None |
| 23 | Patrignani et al, 2021 | 1 | 1 | 1 | 1 | 1 | 1 | 1 | 1 | 8 | Included | None |
| 24 | Sulemankhil et al, 2021 | 1 | 1 | 1 | 1 | 0 | 0 | 1 | 1 | 6 | Included | Clear description of intervention is not present |
| 25 | Garcia et al, 2021 | 1 | 1 | 1 | 1 | 1 | 1 | 1 | 1 | 8 | Included | None |

**Supplementary table_3:Methodological Assessment of Included Original Articles(Cohorts)**

|  | **Selection** |  |  |  | **Comparability** | **Outcome** |  |  | **Quality** |
| --- | --- | --- | --- | --- | --- | --- | --- | --- | --- |
| Study Author, year | Representation of exposed cohort | Selection of non-exposed cohort | Ascertainment of exposure | Demonstration that outcome of interest was not present at start of study | Comparability of cohorts on the basis of design and analysis | Assessment of Outcome | Was follow-up long enough for outcomes to occur | Adequecy of follow-up of cohorts |  |
| Kim *et al,* 2021 | ☆ | 0 | ☆ | ☆ | 0 | ☆ | ☆ | ☆ | Moderate |
| Witberg *et al,*2021 | ☆ | 0 | ☆ | ☆ | 0 | ☆ | ☆ | ☆ | Moderate |
| Diaz *et al*, 2021 | ☆ | 0 | ☆ | ☆ | 0 | ☆ | ☆ | ☆ | Moderate |
| Mevorach *et al,*2021 | ☆ | ☆ | ☆ | ☆ | ☆☆ | ☆ | ☆ | ☆ | High |
| Barda *et al,* 2021 | ☆ | ☆ | ☆ | ☆ | ☆☆ | ☆ | ☆ | ☆ | High |
